# A reversible water‐based electrostatic adhesive

**DOI:** 10.1002/anie.202310750

**Published:** 2023-11-10

**Authors:** Adriana Sierra‐Romero, Katarina Novakovic, Mark Geoghegan

**Affiliations:** ^1^ School of Engineering Newcastle University Newcastle Upon Tyne NE1 7RU UK

**Keywords:** Adhesion, Emulsion Polymers, Interfaces, Polyelectrolytes

## Abstract

Commercial adhesives typically fall into two categories: structural or pressure sensitive. Structural glues rely on covalent bonds formed during curing and provide high tensile strength whilst pressure‐sensitive adhesives use physical bonding to provide weaker adhesion, but with considerable convenience for the user. Here, a new class of adhesive is presented that is also reversible, with a bond strength intermediate between those of pressure‐sensitive and structural adhesives. Complementary water‐based formulations incorporating oppositely charged polyelectrolytes form electrostatic bonds that may be reversed through immersion in a low or high pH aqueous environment. This electrostatic adhesive has the advantageous property that it exhibits good adhesion to low‐energy surfaces such as polypropylene. Furthermore, it is produced by the emulsion copolymerization of commodity materials, styrene and butyl acrylate, which makes it inexpensive and opens the possibility of industrial production. Bio‐based materials have been also integrated into the formulations to further increase sustainability. Moreover, unlike other water‐based glues, adhesion does not significantly degrade in humid environments. Because such electrostatic adhesives do not require mechanical detachment, they are appropriate for the large‐scale recycling of, e.g., bottle labels or food packaging. The adhesive is also suitable for dismantling components in areas as varied as automotive parts and electronics.

## Graphical abstract

A reversible water‐based electrostatic adhesive has been developed through conventional emulsion polymerization using commodity materials. It is made of two formulations, one containing a polycation, and other a polyanion. Detachment is only promoted in highly acidic or alkaline solutions as polyelectrolytes show distinctive charged states. This kind of adhesive would be useful for the recycling and dismantling of products.

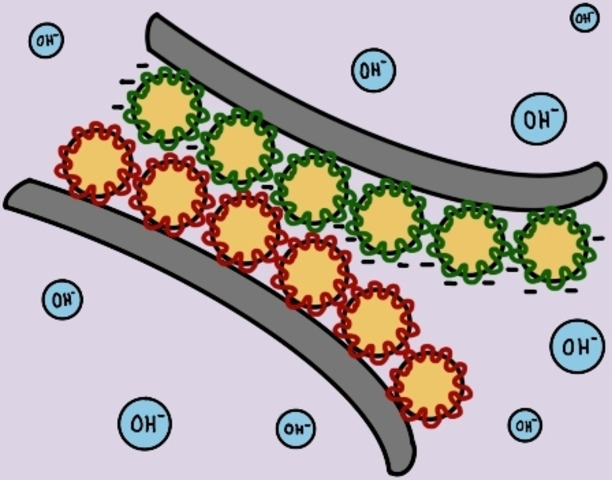

## Introduction

The development of reversible adhesives, which are triggered by a change in temperature,^[1]^ hydration,^[2]^ an electrical potential,^[3]^ exposure to radiation,^[4]^ a magnetic field,^[5]^ or a change in pH,^[6]^ are of interest from both scientific and industrial perspectives.[^7^, ^8^, ^9^] The mechanism of debonding for each case depends on the interfacial chemistry between two surfaces, which very often precludes their integration into large scale industrial processes.

Among these reversible systems, those based on pH changes rely on the interaction between oppositely charged polyelectrolytes in water through electrostatic forces,^[10]^ which does not suffer the inconvenience of requiring mechanical detachment or a source of external fields. Layers of polycations and polyanions have long been known to adhere in water,^[11]^ but the use of weak polyelectrolytes offers a pH‐dependent conformational shape transition that can be leveraged to control adhesion.^[6]^ Weak polycations lose their charges in an alkaline environment, which causes bond failure with the polyanionic component. The converse is true in acidic media. This phenomenon presented the enticing possibility of a viable reversible adhesive system for recycling applications, but an approach that did not place significant demands on the end‐user has been hitherto unavailable.

Emulsion polymerization allows the production of water‐based cationic and anionic adhesive formulations that are scalable and easy to apply. In this work, an electrostatic reversible adhesive system based on complementary cationic and anionic formulations from chitosan and poly(acrylic acid) was developed. Here, reversibility was shown to occur in either high or low pH media. The versatility, ease of production, and low cost of this new kind of adhesive promises to open new opportunities in industries where large‐scale recycling is hindered by the current irreversible bonding of dissimilar components.^[12]^

## Results and Discussion

### Electrostatic reversible adhesion

Emulsion polymerization produces polymer nano‐ or microparticles formed of two phases: a hydrophobic core stabilized by a surfactant shell. In this research, styrene (St) and butyl acrylate (BA) were copolymerized through a free radical process to produce anionic and cationic emulsions depending on the material acting as surfactant. Anionic emulsions were produced with sodium dodecyl sulfate (SDS) as initial stabilizer and by polymerizing acrylic acid as a shell in a second reaction. Cationic emulsions were produced by using chitosan (Chi) as surfactant. It was also demonstrated that styrene can be substituted by acrylated epoxidized soybean oil (AESO), enhancing the sustainable aspect of these materials. Fourier transfer infrared spectroscopy (FTIR) analysis was performed to monitor the polymerization process; relevant spectra are included in Figure S1. Briefly, for St‐based emulsions, spectra at *t*=0 h are predominantly related to the surfactant, as St and BA easily volatilize. As the monomers polymerize, the peaks associated to P(St‐BA) become visible. Something similar occurs for AESO‐based samples, except that AESO is non‐volatile so the bands associated to it are detectable from *t*=0 h. Monomer conversion percentages and solid weight fractions were calculated gravimetrically at *t*=5 h and are presented in Table 1. As AESO is non‐volatile, monomer conversion for AESO‐based formulations only considers BA conversion. It is presumed that agitation (i.e., speed and method) and viscosity are the limiting factors for both monomer conversion and maximum solid content. In particular, the highly viscous chitosan 1.5 wt % solution restricts the diffusion of monomers and initiator, especially during the first stages of polymerization. In addition, the total volume and native viscosity of the monomers contribute to this effect.

**Table 1:** Monomer conversion percentage and solid mass fraction values.

Formulation	Monomer conversion (%)	Solid mass fraction
P(St‐BA)/Chi	74.2±3.8	0.213±0.011
P(St‐BA)/PAA	98.0±3.0	0.288±0.012
P(AESO‐BA)/Chi	87.2±3.2	0.203±0.010
P(AESO‐BA)/PAA	86.6±4.0	0.217±0.010

**Figure 1:**
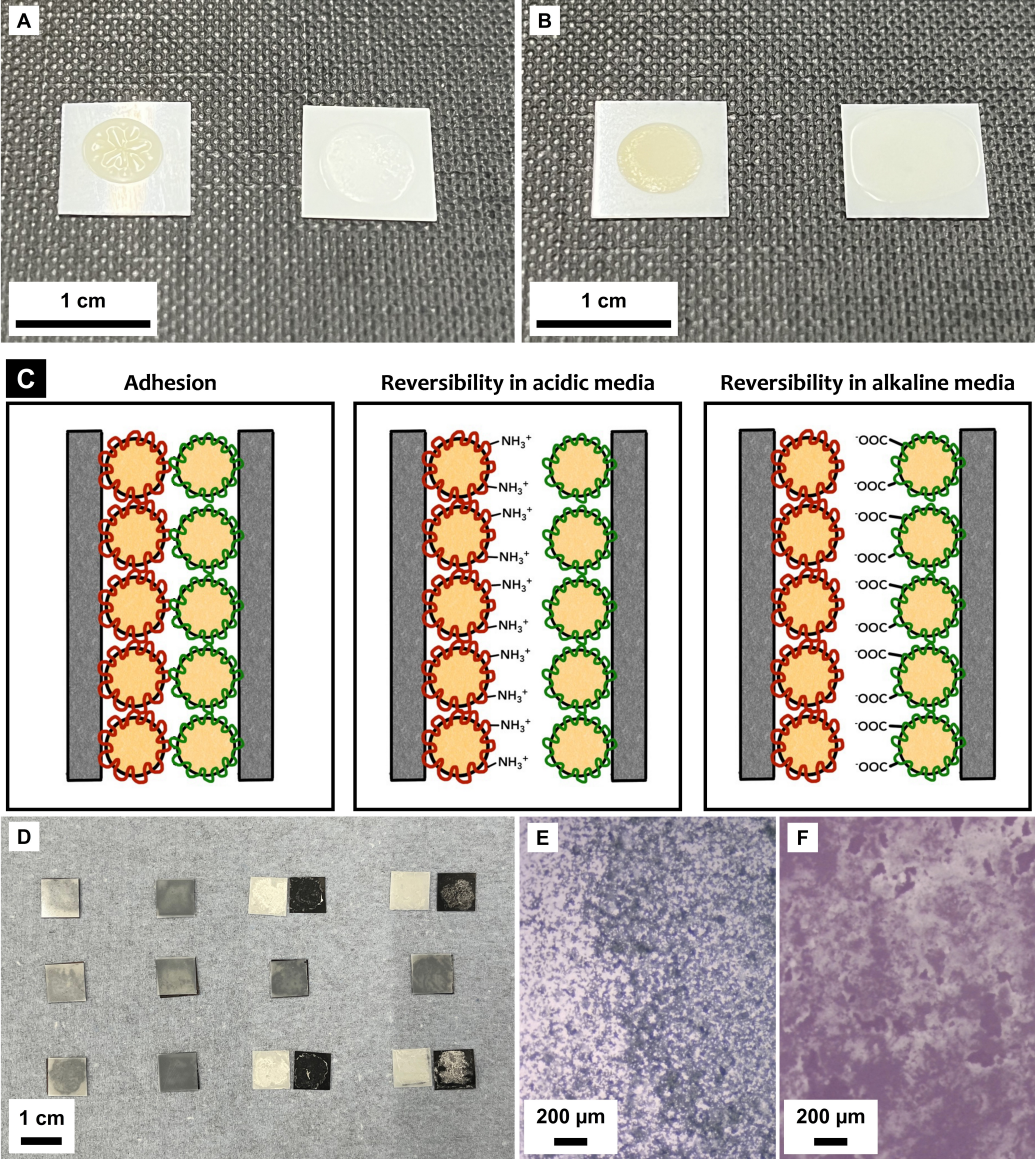
A) P(St‐BA)/Chi (left) and P(St‐BA)/PAA (right) films; B) P(AESO‐BA)/Chi (left) and P(AESO‐BA)/PAA (right) films; C) Proposed detachment mechanism under high and low pH conditions; D) Representative samples showing the reversible behaviour of St‐based adhesives. Top row pH=1, middle row pH=7, bottom row pH=14. From left to right: P(St‐BA)/Chi, P(St‐BA)/PAA, P(St‐BA)/Chi—P(St‐BA)/PAA, and P(St‐BA)/PAA—P(St‐BA)/Chi; E) Representative detached PET surface with P(St‐BA)/Chi; F) Representative detached PP surface with P(St‐BA)/PAA.

For St‐BA formulations, those containing 2 : 3 styrene to butyl acrylate mass ratio were chosen for further study due to their excellent film formation properties. Figure S2 shows representative films produced from the dispersions of the different St‐BA mass ratios. Films form as the charged particles are mechanically forced into close contact which causes counterion condensation, neutralizing monomers beneath the surface of the coating. Formulations resulted in paint‐like viscous liquids that can be easily spread on various surfaces such as: poly(ethylene terephthalate) (PET), polypropylene (PP), cellulose acetate, and glass. Coverage values for each of the previous surfaces and formulations can be found in Table S1. Good film formation at room temperature requires low glass transition temperatures, *T*_g_,^[13]^ which are reported in Figure S3. Here, *T*_g_ values are reported for the St‐BA or AESO‐BA copolymer regions, and the Chi or poly(acrylic acid) (PAA) shells. For instance, Figure 1(A, B) shows the films formed on PET.

To test the reversible behaviour of these adhesive systems, PET and PP were chosen to simulate the application of PP labels to PET bottles. Surfaces measuring 1 cm^2^ were covered with either the anionic or the cationic emulsion, brought together and left to dry. Samples were then exposed to HCl or NaOH solutions with pH ranging from 1 to 14. Table S2 lists the samples that were used for this test. It was found that samples where surfaces were covered with complementary formulations detached overnight when exposed to highly acidic (pH<3) or highly basic (pH>12) solutions. Detachment was observed at the interface between the two layers, i.e., adhesive failure was observed and samples with only cationic or only anionic emulsions did not debond, hence confirming the reversibility of the adhesion.

It is hypothesized that bonding and debonding of this reversible system is based on an analogue mechanism to the one previously reported.[^6^, ^10^, ^14^, ^15^] Cationic emulsions consist of core–shell particles where the hydrophobic core is responsible for the bulk film formation while the surrounding Chi is responsible for the reversible behaviour. Chi is a polysaccharide containing amine functional groups, which can be protonated in acidic environments. Above the p*K*_b_ (≈6.5), depending on the deacetylation degree,^[16]^ the amino groups display no charge (−NH_2_). When exposed to low pH, amine groups become protonated (−NH_3_^+^) and Chi undergoes a conformational change^[17]^ that causes chain expansion. PAA, on the other hand, contains negatively charged carboxylic groups (−COO^−^) above its p*K*_a_ (≈4.5). When exposed to low pH, these groups are neutralized.^[18]^ Relevant zeta potential measurements for the formulations are included in Table S3.

When a surface coated with the anionic emulsion is brought into contact with a cation‐covered one, electrostatic adhesion occurs, and adhesion is strengthened as the system dries. Cationic samples are prepared from an initial acetic acid solution at pH=4.5 so pH<p*K*_b_, and for anionic samples the pH>p*K*_a_, where the initial SDS solution is at pH=6. This locates both formulations in a mid‐pH region where both emulsions carry significant charge, allowing the electrostatic interaction.

When exposed to pH<3, PAA is sufficiently neutralized that the electrostatic bonding fails. In contrast, at pH>12, Chi becomes neutral causing adhesion to fail. Adhesion remains when exposed to neutral conditions, which means the adhesion does not degrade in humid environments. The symmetry of the adhesive failure and the lack of an obvious hydrogen bonding mechanism dictates that the bonding is primarily electrostatic.^[14]^ Figure 1(C) illustrates the proposed adhesion and detachment mechanisms.

The swelling capabilities of individual films were studied in three media at room temperature (20 °C): water, HCl solution at pH=1, and NaOH solution at pH=14. The swelling equilibrium ratio after 24 h was calculated for each sample and it was found that films from cationic emulsions display higher swelling ratios in acidic environment, while films from anionic emulsions possess improved swelling capabilities in alkaline media. Table S4 records the equilibrium swelling ratios for the formulations. In addition, contact angles for liquid droplets on emulsion films were obtained and are summarized in Table 2. Anionic films display the lowest contact angles for alkaline solution and cationic films for acidic solution, further confirming the pH‐dependent hydration processes. Figure S4 presents the relevant images.

**Table 2:** Contact angles on emulsion films for HCl solution pH=1, water and NaOH solution pH=14.

Formulation	HCl solution	Water	NaOH solution
P(St‐BA)/Chi	54.2°	69.3°	76.7°
P(St‐BA)/PAA	53.3°	39.4°	26.4°
P(AESO‐BA)/Chi	60.4°	91.4°	113.3°
P(AESO‐BA)/PAA	55.1°	38.6°	21.2°

The difference in contact angles in contrasting pH environments provides an indication of the debonding mechanism, where Chi leads the hydration and ion diffusion processes at low pH and PAA, at high pH. Detachment is regulated by two main factors. The first of these is the diffusion of the aqueous media and hydration of the films that is hindered by the lack of porosity of the films and their predominantly hydrophobic nature. The second is the concentration of amine groups in Chi or carboxylic groups in PAA. A greater number of functional groups implies the potential for a stronger interaction between systems (more electrostatic bonds). It is worth noticing the hydrophobic nature of cationic films, where the chitosan backbone seemingly confers hydrophobicity to the overall structure. Surface energies for all films were calculated from their contact angles and are reported in Table S5.

Presumably, detachment times can be tuned by modifying the surface contact area through pattering techniques or modifying the chemical identity of either or both polyelectrolytes to reduce the strength of the electrostatic interaction. A second reversibility experiment showed that patterned deposition can be used to decrease detachment times, and the results verifying it are shown in the Supporting Information (Figure S5, Table S6).

### Performance of water‐based reversible adhesives

The performance of the adhesive formulations was investigated in their wet state using a mechanical tester and in their dry state through lap shear measurements. Overall, the strength of adhesion is dependent on the water content at the time of measurement and the mechanisms of adhesion between anionic or cationic emulsions and surface, whereas the film properties are predominantly determined by the presence of either AESO or St.

The strength of adhesion in the wet state was measured for pairs of 1 cm^2^ PET films covered with the same formulation as control or complementary emulsions. In a typical test, a cylindrical probe to which a PET film was attached, approaches the bench surface with a similar PET film on it. Each PET film was covered with the corresponding formulation and the force required for the probe to detach was recorded. A baseline was generated from the test using water to discard any cohesion effects. Figure S6 shows the force of adhesion of individual P(St‐BA)/Chi and P(St‐BA)/PAA couples, and for the P(St‐BA)/Chi—P(St‐BA)/PAA dual system 1 min after having been pressed together with a force of 0.5 N.

The forces of adhesion for the P(St‐BA)/Chi, P(St‐BA)/PAA, and P(St‐BA)/Chi—P(St‐BA)/PAA couples were 0.75 N±0.13 N, 2.48 N±0.38 N and 2.94 N±0.23 N, respectively. Overall, the low values are due to the high‐water content of the as‐prepared emulsions. Although the solid content is lower than commercial adhesive formulations, which contain higher polymer concentrations and additives, the adhesive can keep the joints together until drying consolidates adhesion. Wet adhesion for AESO‐derived formulations was too low to be measured accurately with the available equipment.

**Figure 2:**
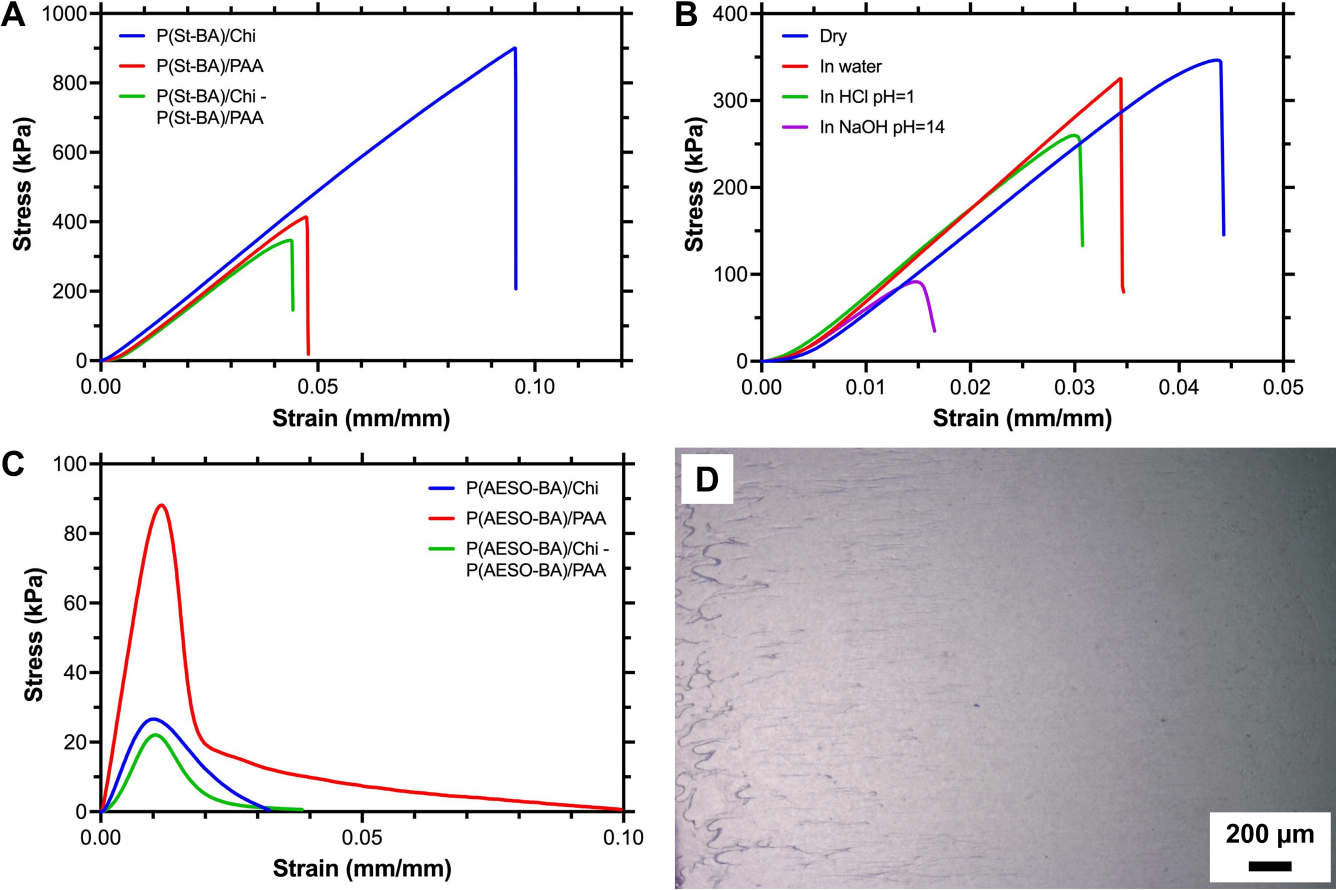
A) Representative stress‐strain curves for St‐based adhesives; B) Representative stress‐strain curves for St‐based samples after they were exposed to water, acid, and alkaline solutions; C) Representative stress‐strain curves for AESO‐based adhesives; D) Optical micrograph of a representative P(AESO‐BA)/PAA sample showing signs of dissipative failure.

Adhesion in the dry state greatly improves for control and dual system samples. Lap shear strength was measured through tension tests on PET films. Figure 2(A) presents the stress‐strain curves for St‐based formulations; curves for AESO‐based emulsions are shown in Figure 2(C). Samples where both PET substrates were covered with P(St‐BA)/Chi have a shear strength of up to 0.91±0.08 MPa, only P(St‐BA)/PAA achieves 0.40±0.02 MPa, and the dual system, 0.36±0.03 MPa. These values position this reversible adhesive in the range between conventional pressure sensitive (tens of kPa) and structural adhesives (typically >5 MPa, depending on the surface).

The shear strengths are linked to the adhesion mechanism that dominates each system and to the native mechanical properties of their components. Polystyrene is a rigid material,^[19]^ but, in contrast, poly(butyl acrylate) is considered soft and commonly used to improve toughness.^[20]^ The properties of the copolymer derived from these are defined by the mass ratio between St and BA, with high St content copolymers being predominantly stiff and high BA content materials, tough.

Although these emulsions contain 2 : 3 St to BA weight ratio, and BA properties are expected to dominate, one must also consider the presence of Chi or PAA; pure Chi and PAA films are known to be brittle.[^21^, ^22^] Both formulations contain a combination of stiff and soft components, although PAA tends to be hygroscopic,^[23]^ which may explain the reduced shear strength for anionic formulations and the dual adhesive system. Overall, water may have a plasticizer effect, so water content in samples used for the lap shear strength tests was measured gravimetrically. For all samples, water content was ∼3 wt %.

An additional lap shear measurement was performed for adhesion to PP films, showing outstanding results (Figure S7, Table S7). In particular, P(St‐BA)/Chi is notable because its shear strength achieves 0.64 MPa±0.02 MPa. PP is a difficult substrate to coat due to its low surface energy (34.9 mN m^−1^, Table S5) and requires specialized glues to stick or plasma treatments to render the surface more compatible. Adhesion to low density polyethylene (LDPE), another low surface energy (28.6 mN m^−1^) material, was tested qualitatively. Figure S8 shows two LDPE films glued by P(St‐BA)/Chi over a surface of 3.2 cm^2^ holding a weight of 300 g. Significantly greater tests were limited by the weakening of the LDPE sample as it stretched, which also precluded quantitative lap shear measurements. A commercial cyanoacrylate glue could also support the weight, but the samples glued with P(St‐BA)/Chi remained flexible, whereas the commercial glue enforced a rigid joint (Figure S8).

While St‐based formulations show a predominantly brittle behaviour during shear deformation, emulsions containing AESO significantly differ. Not only are the shear strengths lower (Table S8), but their behaviour is consistent with typical dissipative failure. While optical analysis of the detached substrates revealed the presence of both adhesive and cohesive failure in St‐based samples, similar analysis for AESO samples show characteristics of dissipative processes (Figure 2(D)). Such distinct behaviours rely on the absence of St and the native properties of AESO, which is often used as a plasticizer.^[24]^

The lap shear properties of the dual (oppositely charged) systems were tested after samples were exposed to water, acid, and alkaline environments for 16 h at 20 °C and reported in Table S9. For the St‐based system, shear strengths were reduced by only 14 % in water, but by 34 % in acid conditions and by 70 % in basic media. Measurements for the degraded mechanical properties of AESO formulations were beyond the reliable detection limits of the equipment and are not included here.

### Stability of water‐based reversible adhesives

Stability of the described formulations was studied for the as‐prepared emulsions over time and for the individual films in different conditions. As‐prepared emulsions were stored at room temperature (16–20 °C) and their particle size measured monthly using light scattering over six months and then after one year. For instance, P(St‐BA)/Chi increased its mean particle size from 306.0 nm to 350.8 nm after six months and to 459.0 nm after one year, P(St‐BA)/PAA, from 122.2 nm to 139.3 nm and then to 178.2 nm over the same time. Table S10 and Figure S9 summarize these findings. Although measurements indicate an increase in mean particle size, the change is not enough to be due to agglomeration or aggregation, demonstrating that the formulations are stable for shelf storage as required for real‐world applications.

**Figure 3:**
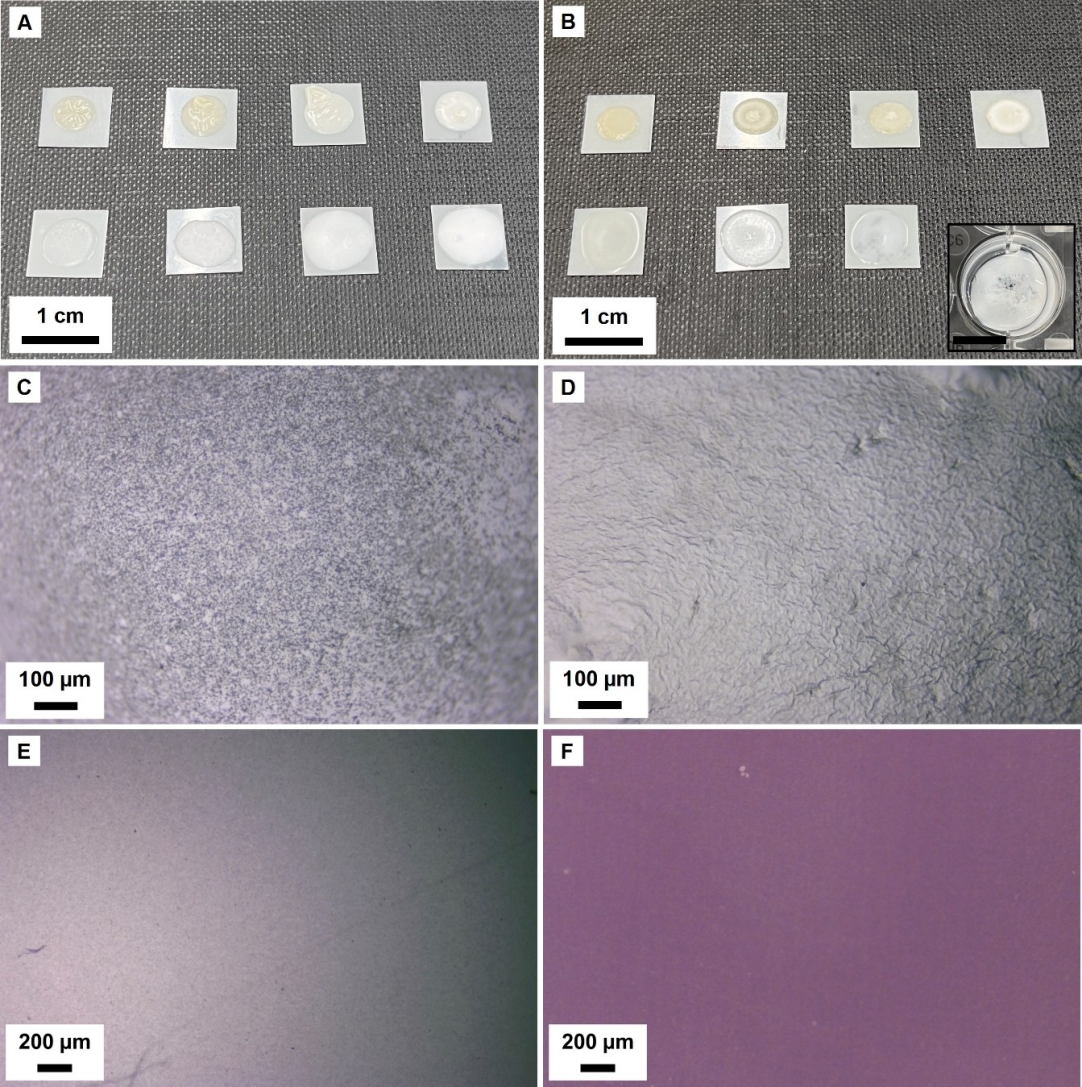
A) (Top, left to right) P(St‐BA)/Chi: As‐prepared film, exposed to water, exposed to acid solution, exposed to alkaline solution. (Bottom, left to right) P(St‐BA)/PAA: As‐prepared film, exposed to water, exposed to acid solution, exposed to alkaline solution. B) (Top, left to right) P(AESO‐BA)/Chi: As‐prepared film, exposed to water, exposed to acid solution, exposed to alkaline solution. (Bottom, left to right) P(AESO‐BA)/PAA: As‐prepared film, exposed to water, exposed to acid solution, exposed to alkaline solution. C) Optical micrograph of as‐prepared P(St‐BA)/Chi film; D) Optical micrograph of as‐prepared P(St‐BA)/PAA film; E) Optical micrograph of PET cleaned with turpentine; F) Optical micrograph of PP cleaned with turpentine.

The ability to recycle components after detachment demands that the previously adhered surfaces are clean. It is therefore important that the coatings can be readily removed after separation. The stability of the individual films was assessed by exposing them to six environments and monitoring their appearance for 30 days to explore meaningful ways the adhesive can be removed after detachment has been promoted. The chosen media were water, acid solution, alkaline solution, ethanol, acetone, and turpentine. Figure 3 shows some images of the films before and after exposure. Additional images are included in Figure S10.

Overall, adhesion of individual films to a PET substrate remains stable in water, acidic, and alkaline media except for P(AESO‐BA)/PAA, which significantly swells in alkaline solution and eventually detaches from the substrate (Figure 3(B)). The resulting film is extremely fragile. St and AESO cationic emulsions in ethanol remained adhered for the duration of the experiment, but anionic emulsions detached after ethanol dried off, presumably due to drastic dehydration processes. Acetone and turpentine are effective at removing the adhesive film in under 2 h. While the physical integrity is conserved for cationic films (i.e., the film remains visible), some physical degradation is observed (Figure S10(A–D)). Anionic films were completely removed and partially solubilized. Figure 3(E, F) shows micrographs of PET and PP after being cleaned with turpentine.

## Conclusion

In summary, water‐based reversible adhesives can be produced through conventional emulsion polymerization. The two‐part adhesive system possesses the necessary film formation properties and is stable for more than a year. Despite being a water‐based adhesive, adhesion does not fail in humid or wet environments. Good performance of the individual formulations and the reversible system is observed intermediate between commercial pressure sensitive and structural adhesives with the advantage of being reversible under highly acidic or alkaline conditions as well as being usable on a variety of surfaces, including previously elusive low‐energy polypropylene. After detachment, the coating can be removed using a standard paint thinner, such as turpentine, to prepare the components for recycling.

## Supporting Information

Materials and Methods
Figures S1 to S10
Tables S1 to S10
Movies S1 to S2

## Conflict of interest

UK PCT patent application (P340927GB).

1

## Supporting information

As a service to our authors and readers, this journal provides supporting information supplied by the authors. Such materials are peer reviewed and may be re‐organized for online delivery, but are not copy‐edited or typeset. Technical support issues arising from supporting information (other than missing files) should be addressed to the authors.

Supporting Information

Supporting Information

Supporting Information

## Data Availability

The data that support the findings of this study are available from the corresponding author upon reasonable request.
