# Supplementary material for: A reversible water‐based electrostatic adhesive
Source: Angew Chem Int Ed Engl. 2023 Nov 10;63(2):e202310750. doi: 10.1002/anie.202310750 (PMC10952749; doi:10.1002/anie.202310750)
Supplement: Supplementary file 1 — Supporting Information [file ANIE-63-0-s002.pdf]

Angewandte Chemie
Eine Zeitschrift der Gesellschaft Deutscher Chemiker

Supporting Information

A reversible water-based electrostatic adhesive

*A. Sierra-Romero, K. Novakovic, M. Geoghegan**

Supporting Information
©Wiley-VCH 2023
69451 Weinheim, Germany

A reversible water-based electrostatic adhesive

Adriana Sierra-Romero, Katarina Novakovic, and Mark Geoghegan*

Abstract: Commercial adhesives typically fall into two categories: structural or pressure sensitive. Structural glues rely on covalent bonds formed during curing and provide high tensile strength whilst pressure-sensitive adhesives use physical bonding to provide weaker adhesion, but with considerable convenience for the user. Here, a new class of adhesive is presented that is also reversible, with a bond strength intermediate between those of pressure-sensitive and structural adhesives. Complementary water-based formulations incorporating oppositely charged polyelectrolytes form electrostatic bonds that may be reversed through immersion in a low or high pH aqueous environment. This electrostatic adhesive has the advantageous property that it exhibits good adhesion to low-energy surfaces such as polypropylene. Furthermore, it is produced by the emulsion copolymerization of commodity materials, styrene and butyl acrylate, which makes it inexpensive and opens the possibility of industrial production. Bio-based materials have been also integrated into the formulations to further increase sustainability. Moreover, unlike other water-based glues, adhesion does not significantly degrade in humid environments. Because such electrostatic adhesives do not require mechanical detachment, they are appropriate for the large-scale recycling of, e.g., bottle labels or food packaging. The adhesive is also suitable for dismantling components in areas as varied as automotive parts and electronics.

DOI: 10.1002/anie.202310750

Table of Contents

Experimental Procedures ... **3**
Results and Discussion ... **6**
Figure S1 ... 6
Table S1 ... 6
Figure S2 ... 6
Figure S3 ... 7
Table S2. ... 7
Table S3. ... 8
Table S4. ... 8
Table S5. ... 8
Figure S4 ... 9
Figure S5 ... 10
Table S6. ... 10
Figure S6 ... 10
Figure S7 ... 11
Table S7. ... 11
Table S8. ... 11
Table S9. ... 11
Table S10. ... 12
Figure S8 ... 12
Figure S9 ... 13
Figure S10. ... 14
Reference ... **15**
Author Contributions ... **15**

Experimental Procedures

Materials. Sodium dodecyl sulfate (SDS) ($NaC_{12}H_{25}SO_4$, ≥ 98.5%), chitosan (Chi) (medium molar mass), acetic acid (AAc) (CH_3COOH, 99.8-100.5%), styrene (St) (C_8H_8, ≥ 99%), acrylated epoxidized soybean oil (AESO), butyl acrylate (BA) ($C_7H_{12}O_2$, stabilized for synthesis), acrylic acid (AA) ($CH_2CHCOOH$, 99%), and potassium persulfate (KPS) ($K_2S_2O_8$, ≥ 99.0%) were all purchased from Sigma-Aldrich were used as received for the synthesis of the emulsions. Hydrochloric acid (HCl) (37%), sodium hydroxide (NaOH) (≥ 98%), ethanol (absolute for analysis), and acetone (≥ 99.5%) purchased from Sigma-Aldrich were used as received for reversibility, swelling, and film stability tests. Distilled turpentine purchased from Winsor & Newton art supplies was used for film stability tests.
Mylar® plastic films (PET) 0.25 mm thickness and polypropylene (PP) plastic films 0.127 mm thickness were purchased from RS Components and used for the reversibility experiments. Mylar® plastic films (PET) 0.25 mm thickness and polypropylene plastic films 0.45 mm thickness were purchased from RS Components and used for the lap shear strength measurements. Cyanoacrylate Superglue purchased from RS Components and hanging hooks from JELLYSUB were used for the tensile strength demonstrations.

Synthesis of poly(styrene-butyl acrylate)/chitosan emulsion (P(St-BA)/Chi). The synthesis of P(St-BA)/Chi was performed following a typical free-radical emulsion polymerization technique. It was carried out in a 100 mL three-neck flask in an oil bath where 20 mL of a 1.0 wt% AAc solution in deionized water was poured. After setting the stirrer at 400 rpm, 0.3 g of Chi were added and dissolved overnight (~15 h). Stirring was increased to 900 rpm as a mixture of St and BA (8.1 g of monomers at 3:2, 1:1, and 2:3 St:BA mass ratio) was added dropwise during 10 min and the nitrogen flow was turned on. The emulsion was left to develop for 15 min before adding 40 mg of KPS dissolved in 1 mL of deionized water and turning the heating on to 70 °C. As the temperature reached 70 °C, stirring was lowered to 500 rpm, the nitrogen flow was minimized, and the reaction was left to proceed for 5 h. The resulting emulsion was collected in a vial and stored for analysis.

Synthesis of poly(styrene-butyl acrylate)/poly(acrylic acid) emulsion (P(St-BA)/PAA). The synthesis of P(St-BA)/PAA was performed following a typical free-radical emulsion polymerization technique. It was carried out in a 100 mL three-neck flask in an oil bath where 20 mL of deionized water was poured. After setting the stirrer at 350 rpm, 0.3 g of SDS were added and dissolved for 10 min. Then, a mixture of St and BA (8.1 g of monomers at 3:2, 1:1, and 2:3 St:BA mass ratio) was added dropwise during 5 min and the nitrogen flow was turned on. The emulsion was left to develop for 15 min before adding 40 mg of KPS dissolved in 1 mL of deionized water and turning the heating on to 70 °C. The nitrogen flow was lowered as the temperature reached 70 °C and the reaction was left to proceed for 2 h. After that time, 10 mg of KPS dissolved in 0.5 mL of deionized water were added dropwise, followed by 1 mL of AA in 1 mL of deionized water, dropwise over 5 min. Polymerization proceeded for further 3 h and the resulting emulsion was collected in a glass vial and stored for analysis.

Synthesis of poly(acrylated epoxidized soybean oil-butyl acrylate)/chitosan emulsion (P(AESO-BA)/Chi). The synthesis of P(AESO-BA)/Chi was performed following a typical free-radical emulsion polymerization technique. It was carried out in a 100 mL three-neck flask in an oil bath where 20 mL of a 1.0 wt% AAc solution in deionized water was poured. After setting the stirrer at 400 rpm, 0.3 g of Chi were added and dissolved overnight (~15 h). Stirring was increased to 900 rpm as AESO dissolved in BA (5.97 g of monomers at 1:3 AESO:BA mass ratio) was added dropwise during 10 min and the nitrogen flow was turned on. The emulsion was left to develop for 15 min before adding 20 mg of KPS dissolved in 1 mL of deionized water and turning the heating on to 70 °C. As the temperature reached 70 °C, stirring was lowered to 450 rpm, the nitrogen flow was minimized, and the reaction was left to proceed for 5 h. The resulting emulsion was collected in a vial and stored for analysis.

Synthesis of poly(acrylated epoxidized soybean oil-butyl acrylate)/poly(acrylic acid) emulsion (P(AESO-BA)/PAA). The synthesis of P(AESO-BA)/PAA was performed following a typical free-radical emulsion polymerization technique. It was carried out in a 100 mL three-neck flask in an oil bath where 20 mL of deionized water was poured. After setting the stirrer at 350 rpm, 0.3 g of SDS were added and dissolved for 10 min. Then, AESO dissolved in BA (5.97 g of monomer at 1:3 AESO:BA mass ratio) was added dropwise during 5 min and the nitrogen flow was turned on. The emulsion was left to develop for 15 min before adding 20 mg of KPS dissolved in 1 mL of deionized water and turning the heating on to 70 °C. The nitrogen flow was lowered as the temperature reached 70 °C and the reaction was left to proceed for 2 h. After that time, 10 mg of KPS dissolved in 0.5 mL of deionized water were added dropwise, followed by 1 mL of AA in 1 mL of deionized water, dropwise over 5 min. Polymerization proceeded for further 3 h and the resulting emulsion was collected in a glass vial and stored for analysis.

Film formation and coverage. Film formation properties were studied by casting the prepared emulsion on PET, PP, glass, and cellulose acetate substrates at room temperature (20 °C) previously wiped with ethanol. Coverage was calculated by weighing the emulsion required to cover a defined area on each substrate. Coverage tests were performed in triplicate.

Monomer conversion. Monomer conversion was evaluated through a gravimetric test. A small amount of emulsion was drawn from the reactor flask at t = 0 h – 5 h, put in a silicone container, weighed, and dried in a fume hood. The total monomer conversion was calculated by the gravimetric method using

$$\text{Conversion} = \frac{W_{\text{dry}} - W_{\text{wet}} \times (\text{S+KPS})\text{wt\%}}{W_{\text{wet}} \times \eta},$$

where W_{dry} is the mass of the residue in the weighing plate; $(\mathrm{S}+\mathrm{KPS})\mathrm{wt}\%$ is the total mass percentage of S (surfactant) and initiator in the reactant mixture; W_{wet} is the mass of the emulsion content drawn to the weighing pan; and η is the mass percentage of monomers in the entire reactant mixture. This was done for three independent reactions and a mean value calculated.

Solid fraction. The solid fraction of each emulsion was determined by drying a weighed emulsion sample (W_0) for 1 h at 100 °C. The dry residue (W_1) was then weighed. This was done in triplicate for three independent reactions. The solid fraction was calculated using

$$\text{Solid fraction} = \frac{W_1}{W_0}.$$

Attenuated total reflection infrared spectroscopy (ATR-FTIR). ATR-FTIR spectra of dry samples were acquired using an Agilent Cary 630 instrument from 450 cm^{-1} to 4000 cm^{-1} taking 64 scans per spectra, with a resolution of 4 cm^{-1}.

Differential scanning calorimetry (DSC). DSC analysis was performed on dried samples using a TA Instruments Q Series differential scanning calorimeter through a heat-cool-heat cycle at a heating rate of 10°C min^{-1} from –50 °C, up to 200 °C under a nitrogen flow of 50 mL min^{-1}. The second heating cycle was used to identify the relevant thermal properties.

Reversibility of adhesion. The reversibility of the adhesive was evaluated at pH = 1, 2…, 13, 14 HCl or NaOH solutions without stirring. Samples were prepared by coating PET and PP films with each of the emulsions, brought in contact, and left to dry at room temperature for 24 h. Samples were immersed in each pH condition at room temperature and adhesion was evaluated periodically. **Table S2** identifies each sample. The test was performed in triplicate.

Force of adhesion. The force of adhesion of the emulsions in the wet state was measured using a Stable Microsystems TA.XTplusC Texture Analyser. It consists of a cylindrical probe that approaches a surface and then measures the force required for the probe to detach. A PET film was attached to the bottom part of the probe and to the surface. For every test, each was covered with either the cationic or anionic emulsions. A control test was first recorded for water to discard possible cohesion effects. Forces of adhesion between cationic or anionic emulsions and themselves were recorded as control. Then, the surface PET was coated with the cationic emulsion, the probe PET with anionic emulsion, and the force of adhesion measured. The probe speed was set at 50 mm min^{-1}, it was held on the surface with a force of 0.5 N for 1 min and then receded at the same speed.

Lap shear tests. Lap shear tests for PET films joined by the prepared emulsions were performed using a Shimadzu tester fitted with a 1 kN load cell at a speed of 1 mm min^{-1}. Samples were prepared according to the following diagram. Five samples for each adhesive system were prepared and the mean shear strength calculated. For St-based emulsions, the test was also performed on PP substrates.

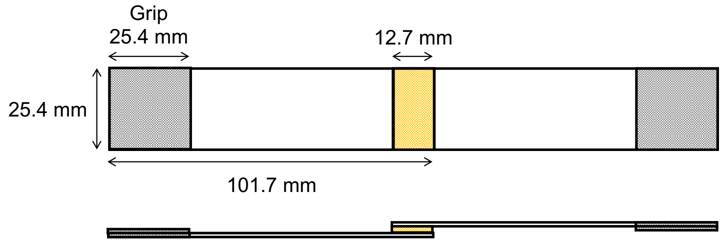

A second batch of samples for the dual adhesive systems (both St and AESO-based) were prepared and immersed in either water, HCl pH = 1 solution or NaOH pH = 14 solution without stirring. The tests were performed after 16 h to record the degradation of the mechanical properties as detachment is promoted. Substrates were wiped with acetone prior adhesive deposition.

Tensile strength demonstration. A demonstration of the tensile strength of adhesion was performed for St-based emulsions. A typical sample was prepared with two PET films measuring 3.2 cm^2 that were adhered to a pair of plastic hooks using a cyanoacrylate glue. After they dried, one was coated with P(St-BA)/Chi, the other with P(St-BA)/PAA and brought together. Demonstrations were performed in the dry state, and after 24 h immersed in either water, HCl pH = 1 solution, or NaOH pH = 14 solution without stirring.

Storage stability. Each emulsion was stored in a glass vial, tightly sealed at room temperature (16 °C – 20 °C) and away from sunlight. Stability was monitored by measuring the particle size by means of light scattering analysis using a Zetasizer Nano. One droplet of the as-prepared emulsion was diluted with 2 mL of water in a polystyrene cuvette. Each measurement was repeated three times, and the mean particle size, reported. The test was repeated each month for six months and then after a year.

Swelling capacity tests. The swelling of emulsion films was evaluated by an immersion assay at room temperature (20 °C) without stirring. Samples were prepared by casting films on a PTFE substrate. Dry samples (W_0) were immersed in deionized water, HCl pH = 1 solution or NaOH pH = 14 solution. After 24 h, the films were removed, wiped with a filter paper to remove excess liquid, and weighed (W_1). Tests were performed in triplicate. The equilibrium swelling ratio (ESR) of the films was calculated using

$$\text{ESR}\ \% = \left(\frac{W_1 - W_0}{W_0}\right) \times 100\,.$$

Contact angle. The contact angles were measured by setting a 10 mL droplet of water, HCl solution pH = 1 or NaOH solution pH = 14 on each emulsion film. A digital photograph of the transverse section was taken, and the angle measured using ImageJ software. The surface energy of the films was calculated from these contact angles.[1]

Chemical resistance and film stability. The chemical resistance of films attached to PET was assessed in different environments to identify the conditions that may lead to detachment of the adhesive from the substrate. Dry films were immersed in either water, HCl pH = 1 solution, NaOH pH = 14 solution, ethanol, acetone, or turpentine without stirring. Their appearance was monitored for 30 days visually and through optical microscopy using a B-150D-MRPL Optika microscope. Tests were performed in duplicate.

Results and Discussion

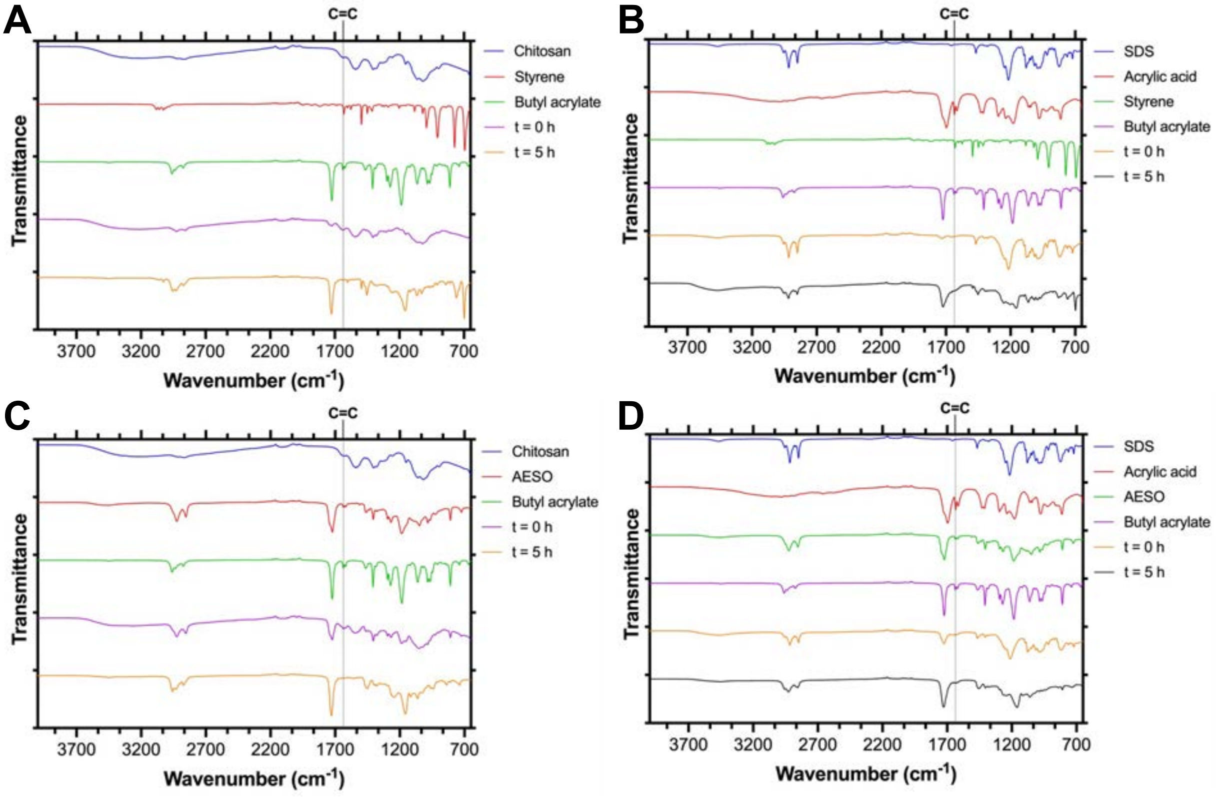

Figure S1. FT-IR spectra of A) P(St-BA)/Chi; B) P(St-BA)/PAA; C) P(AESO-BA)/Chi; D) P(AESO-BA)/PAA.

Table S1. Coverage values in mg cm^{-1}

Formulation	PET	PP	Glass	Cellulose acetate
P(St-BA)/Chi	57.2 ± 0.4	17.8 ± 0.1	7.7 ± 0.1	8.2 ± 0.1
P(St-BA)/PAA	6.3 ± 0.6	9.9 ± 0.3	5.8 ± 0.6	6.4 ± 0.3
P(AESO-BA)/Chi	7.8 ± 0.6	14.1 ± 1.3	6.0 ± 0.6	7.8 ± 0.7
P(AESO-BA)/PAA	8.2 ± 0.7	7.9 ± 0.6	4.4 ± 0.2	3.2 ± 0.3

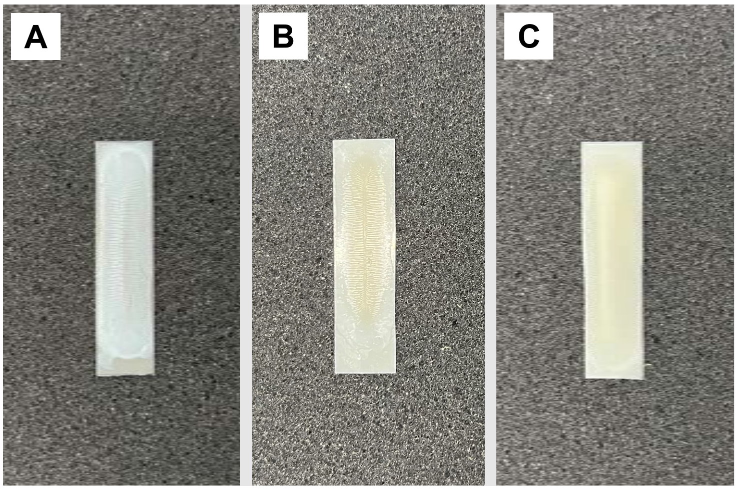

Figure S2. St-based film samples containing A) 3:2 St:BA mass ratio, B) 1:1 St:BA mass ratio, and C) 2:3 St:BA mass ratio. A and B show the formation of cracks.

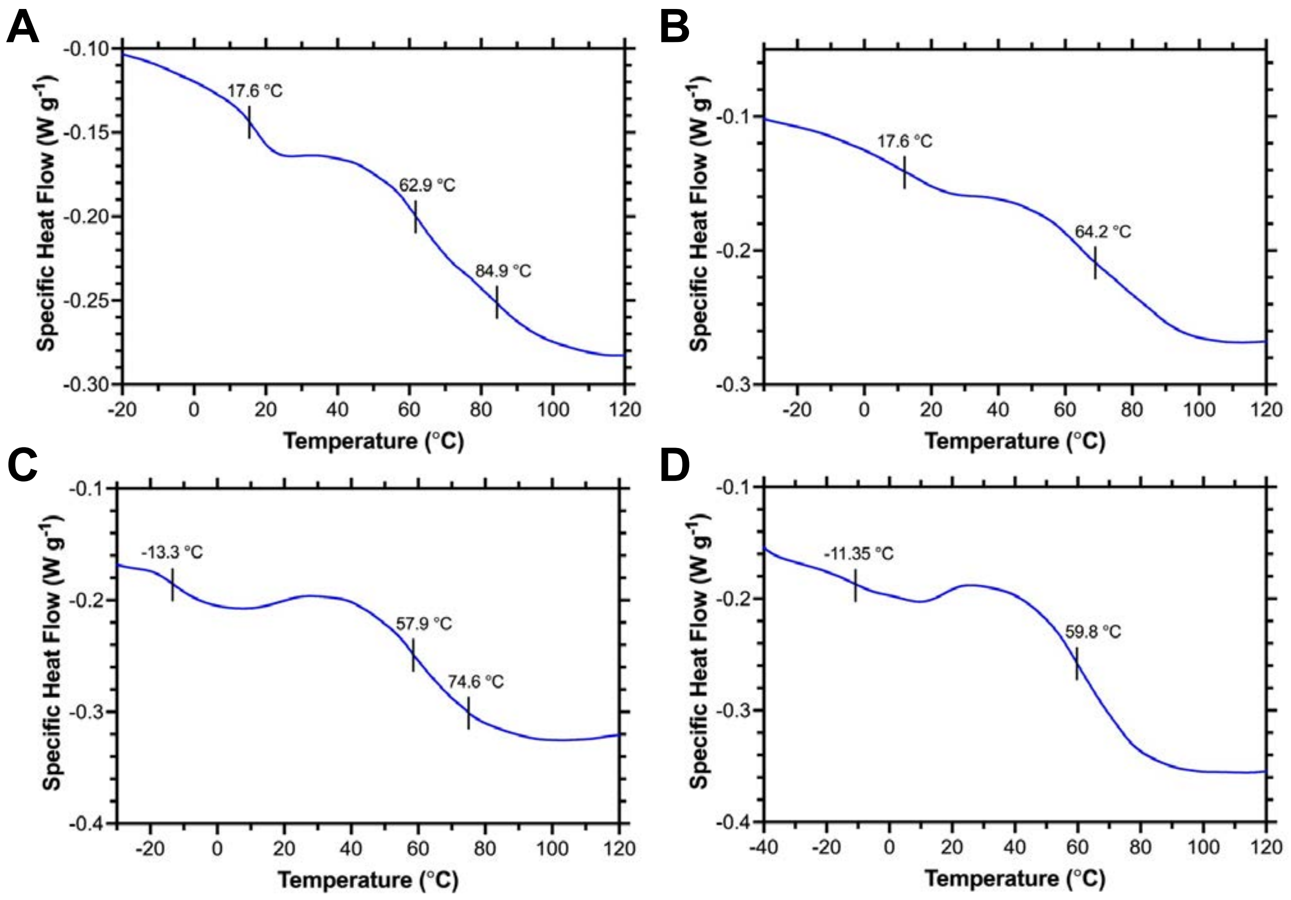

Figure S3. Differential scanning calorimetry thermograms showing the glass transition temperatures of A) P(St-BA)/Chi; B) P(St-BA)/PAA; C) P(AESO-BA)/Chi; D) P(AESO-BA)/PAA.

Table S2. Samples for reversibility test.

Formulation on PET	Formulation on PP
P(St-BA)/Chi	P(St-BA)/Chi
P(St-BA)/PAA	P(St-BA)/PAA
P(St-BA)/Chi	P(St-BA)/PAA
P(St-BA)/PAA	P(St-BA)/Chi
P(AESO-BA)/Chi	P(AESO-BA)/Chi
P(AESO-BA)/PAA	P(AESO-BA)/PAA
P(AESO-BA)/Chi	P(AESO-BA)/PAA
P(AESO-BA)/PAA	P(AESO-BA)/Chi

Table S3. Zeta potential measurements of the emulsions at different pH conditions.

Formulation	In HCl solution pH=1 (mV)	In DW pH=6 (mV)	In NaOH solution pH=14 (mV)
P(St-BA)/Chi	28.5 ± 2.0	33.8 ± 0.2	-0.31 ± 0.58
P(St-BA)/PAA	-5.3 ± 1.5	-32.2 ± 1.1	-34.4 ± 2.0
P(AESO-BA)/Chi	26.3 ± 0.5	35.5 ± 1.0	-1.6 ± 0.3
P(AESO-BA)/PAA	-4.54 ± 0.38	-30.1 ± 0.4	-30.2 ± 2.6

Table S4. Equilibrium swelling ratio after 24 h at room temperature (20 °C).

Formulation	Condition	Equilibrium swelling ratio (%)
P(St-BA)/Chi	Water	4.2 ± 0.4
	HCl solution pH=1	13.8 ± 1.4
	NaOH solution pH=14	4.8 ± 0.4
P(St-BA)/PAA	Water	13.5 ± 1.3
	HCl solution pH=1	13.9 ± 0.7
	NaOH solution pH=14	40.7 ± 3.9
P(AESO-BA)/Chi	Water	5.6 ± 0.6
	HCl solution pH=1	17.2 ± 1.3
	NaOH solution pH=14	6.2 ± 0.6
P(AESO-BA)/PAA	Water	14.6 ± 1.1
	HCl solution pH=1	16.1 ± 1.5
	NaOH solution pH=14	587.6 ± 0.6

Table S5. Calculated surface energy for all films.[1]

Formulation	Surface energy (mN m^{-1})
P(St-BA)/Chi	34.9
P(St-BA)/PAA	61.5
P(AESO-BA)/Chi	27.2
P(AESO-BA)/PAA	60.0

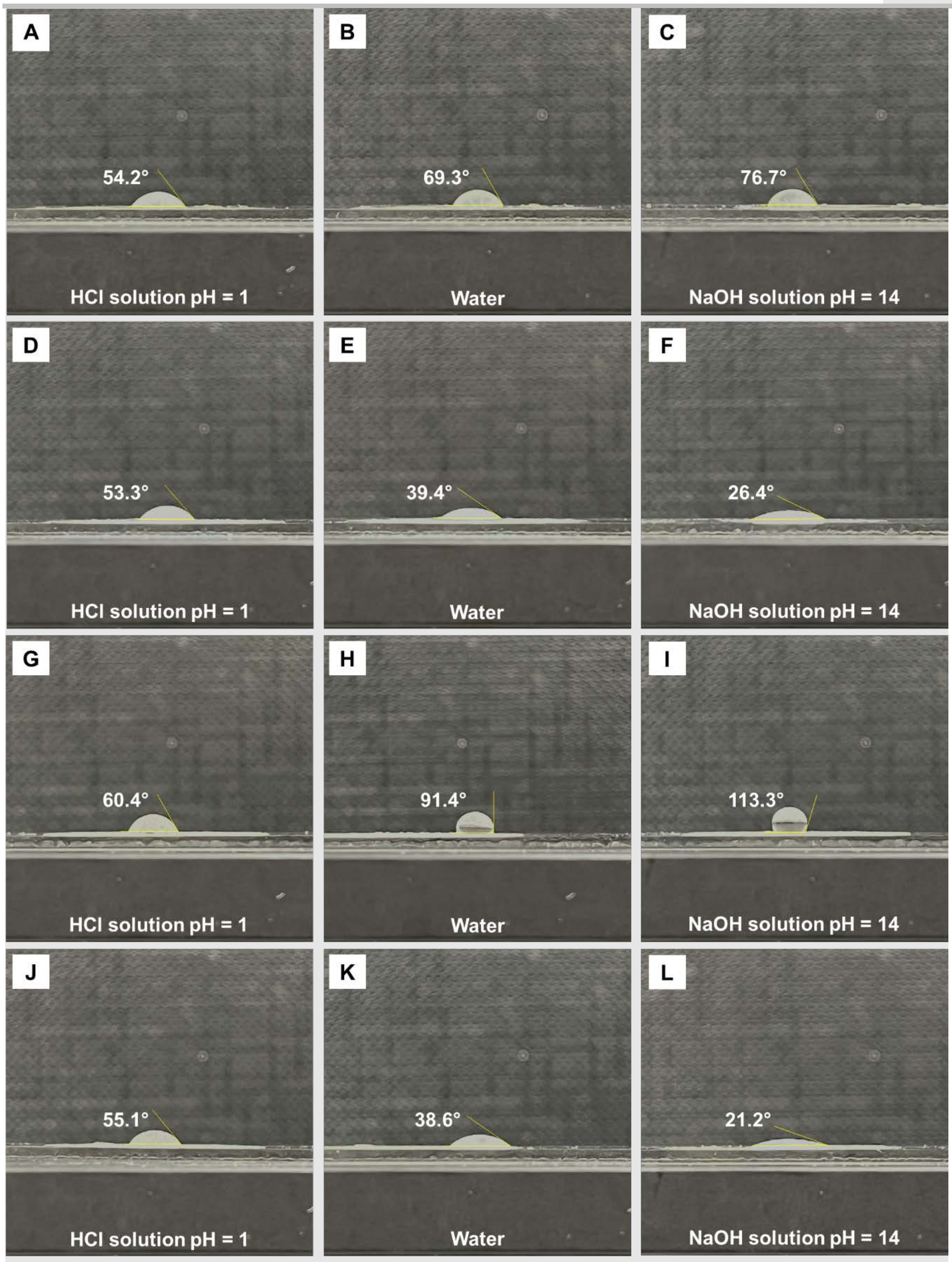

Figure S4. A-C) Contact angles for P(St-BA)/Chi films; D-F) Contact angles for P(St-BA)/PAA films; G-I) Contact angles for P(AESO-BA)/Chi films; J-L) Contact angles for P(AESO-BA)/PAA films.

Five deposition patterns 1-5 were produced on PET films. These are shown in **Fig. S5** where the crosses indicate the positions where the adhesive was deposited; all areas sum up to 1 cm^2. Samples were exposed to pH 1 and 14 solutions and their adhesive stability verified every 30 min. **Table S6** summarizes the recorded detachment times. According to these results, it was demonstrated that detachment times can be greatly improved by creating patterns that maximize the adhesive exposure to the aqueous environment. This behaviour is consistent with the measured swelling ratios, where films from anionic emulsions are capable of absorbing larger amounts of water in basic solutions. It is to be expected that there is scope for further optimization using different shapes and perhaps even topographies.

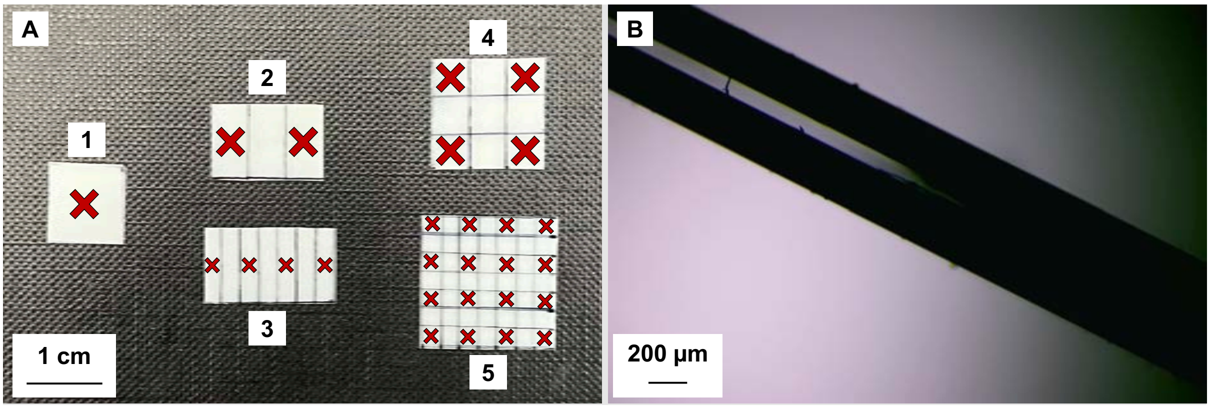

Figure S5. A) Patterned substrates; B) Representative optical micrograph of the transverse view of pattern.

Table S6. Detachment times for different patterns at low and high pH values.

System	Pattern	Detachment time (h) pH=1	Detachment time (h) pH=14
P(St-BA)/Chi - P(St-BA)/PAA	1	Overnight	Overnight
	2	Overnight	9.0
	3	9.5	7.5
	4	10.0	7.5
	5	9.0	8.0
P(AESO-BA)/Chi - P(AESO-BA)/PAA	1	Overnight	Overnight
	2	7.0	4.5
	3	5.0	3.5
	4	5.5	4.0
	5	5.0	4.5

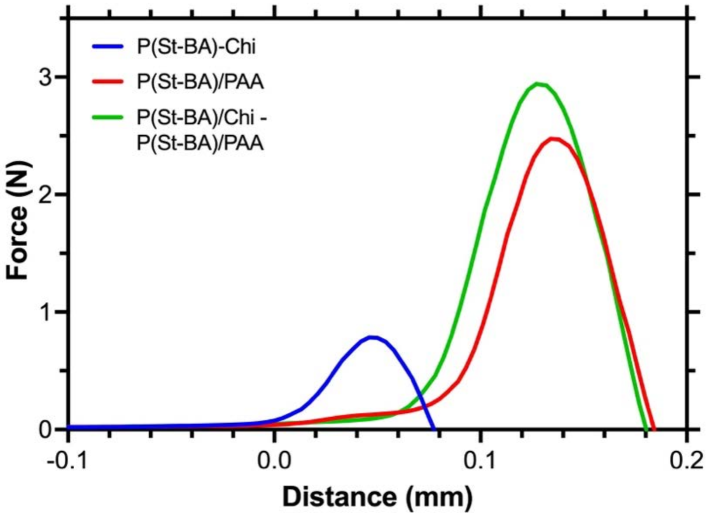

Figure S6. Force of adhesion of wet P(St-BA)/Chi, P(St-BA)/PAA and P(St-BA)/Chi - P(St-BA)/PAA system.

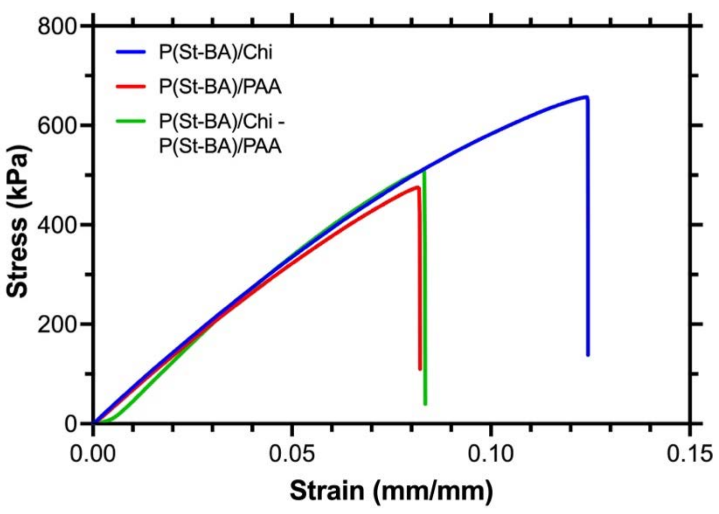

Figure S7. Representative stress vs strain curves for St-based adhesives on PP.

Table S7. Lap shear strengths for St-derived formulations on PP.

Formulation	Lap shear strength (MPa)
P(St-BA)/Chi	0.64 MPa ± 0.02 MPa
P(St-BA)/PAA	0.49 MPa ± 0.04 MPa
P(St-BA)/Chi – P(St-BA)/PAA	0.49 MPa ± 0.06 MPa

Table S8. Lap shear strengths for AESO-derived formulations on PET.

Formulation	Lap shear strength (kPa)
P(AESO-BA)/Chi	28.6 ± 2.1
P(AESO-BA)/PAA	83.8 ± 7.5
P(AESO-BA)/Chi – P(AESO-BA)/PAA	23.6 ± 2.3

Table S9. Lap shear strengths for complementary (dual) St-derived formulations on PET after degradation.

Conditions	Lap shear strength (MPa)
Dry	0.36 ± 0.03
After 16 h in water	0.31 ± 0.03
After 16 h in HCl solution pH=1	0.24 ± 0.02
After 16 h NaOH solution pH=14	0.11 ± 0.01

Table S10. Particle size and polydispersity of as-prepared emulsions, after six months and one year of storage.

	As prepared		After 6 months		After one year	
Formulation	Mean size (d.nm)	PDI	Mean size (d.nm)	PDI	Mean size (d.nm)	PDI
P(St-BA)/Chi	306.0 ± 8.4	0.096	350.8 ± 22.9	0.096	459.0 ± 29.7	0.104
P(St-BA)/PAA	122.2 ± 10.9	0.249	139.3 ± 10.2	0.259	178.2 ± 24.1	0.282
P(AESO-BA)/Chi	342.9 ± 2.3	0.070	351.9 ± 9.6	0.088	420.3 ± 17.5	0.091
P(AESO-BA)/PAA	507.8 ± 70.9	0.278	552.2 ± 41.0	0.280	714.6 ± 37.4	0.286

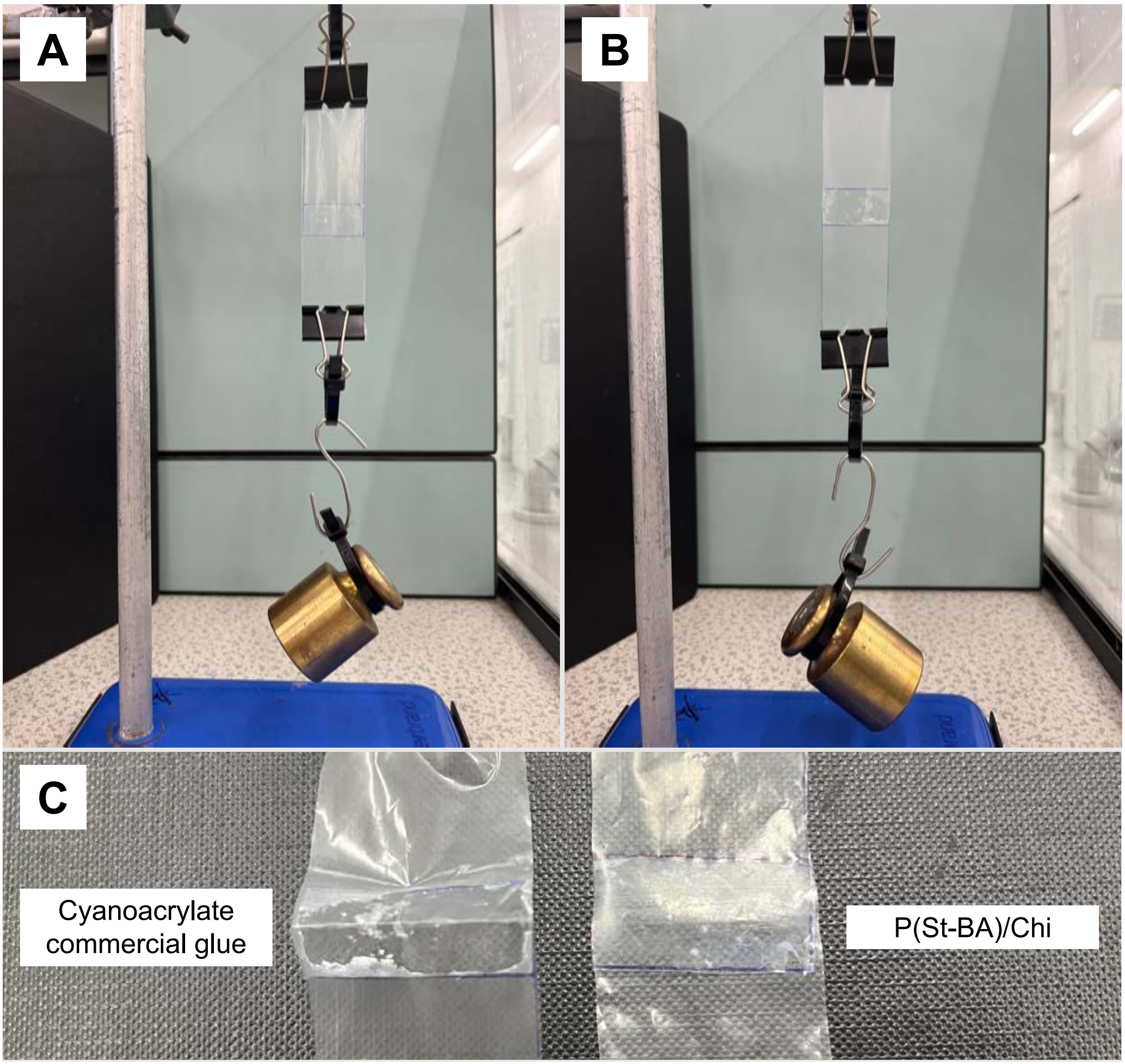

Figure S8. A) LDPE glued with P(St-BA)/Chi holding a 300 g mass; B) LDPE glued with a cyanoacrylate commercial adhesive holding a 300 g mass; C) LDPE films keep their flexibility when glued with P(St-BA)/Chi.

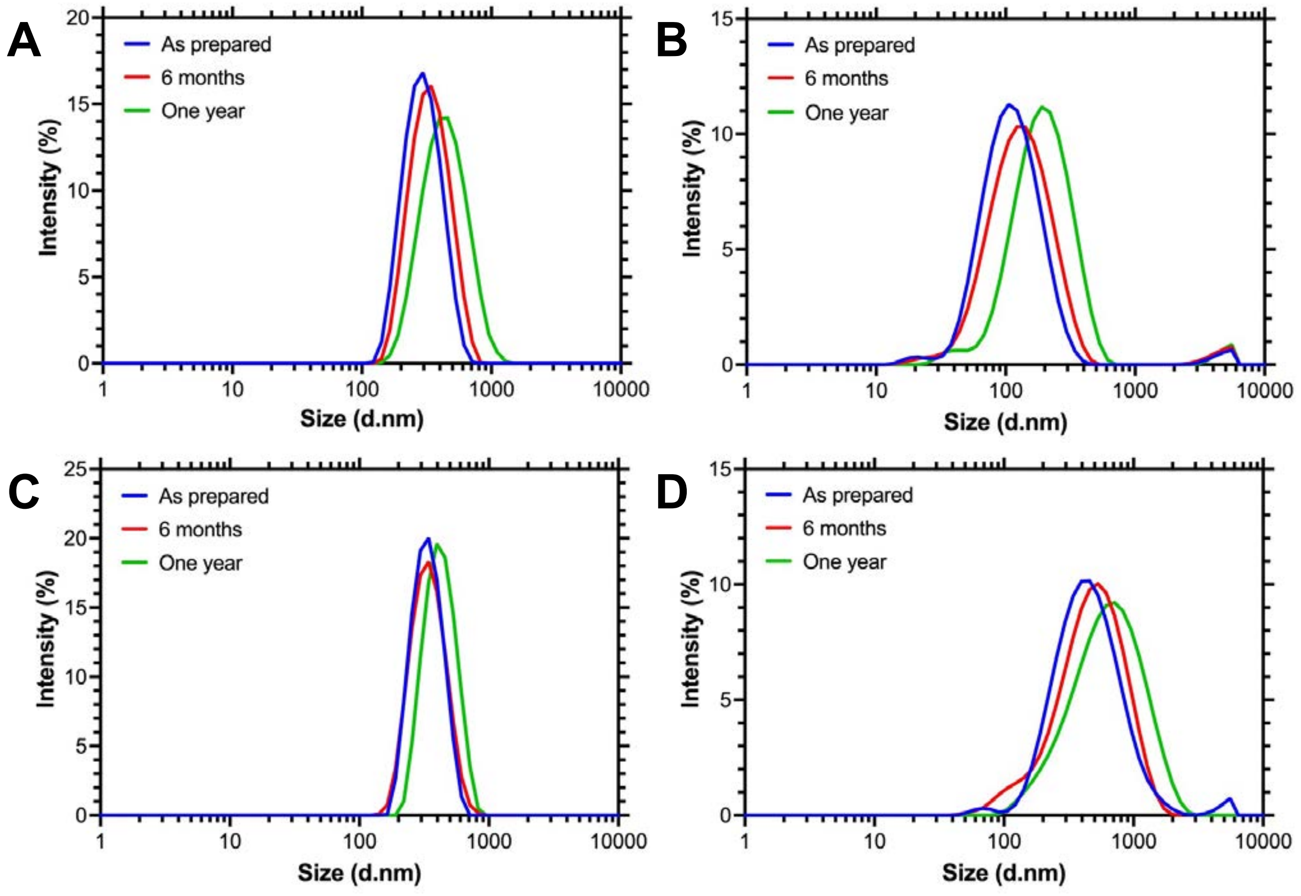

Figure S9. Particle size distribution over time of A) P(St-BA)/Chi; B) P(St-BA)/PAA; C) P(AESO-BA)/Chi; D) P(AESO-BA)/PAA.

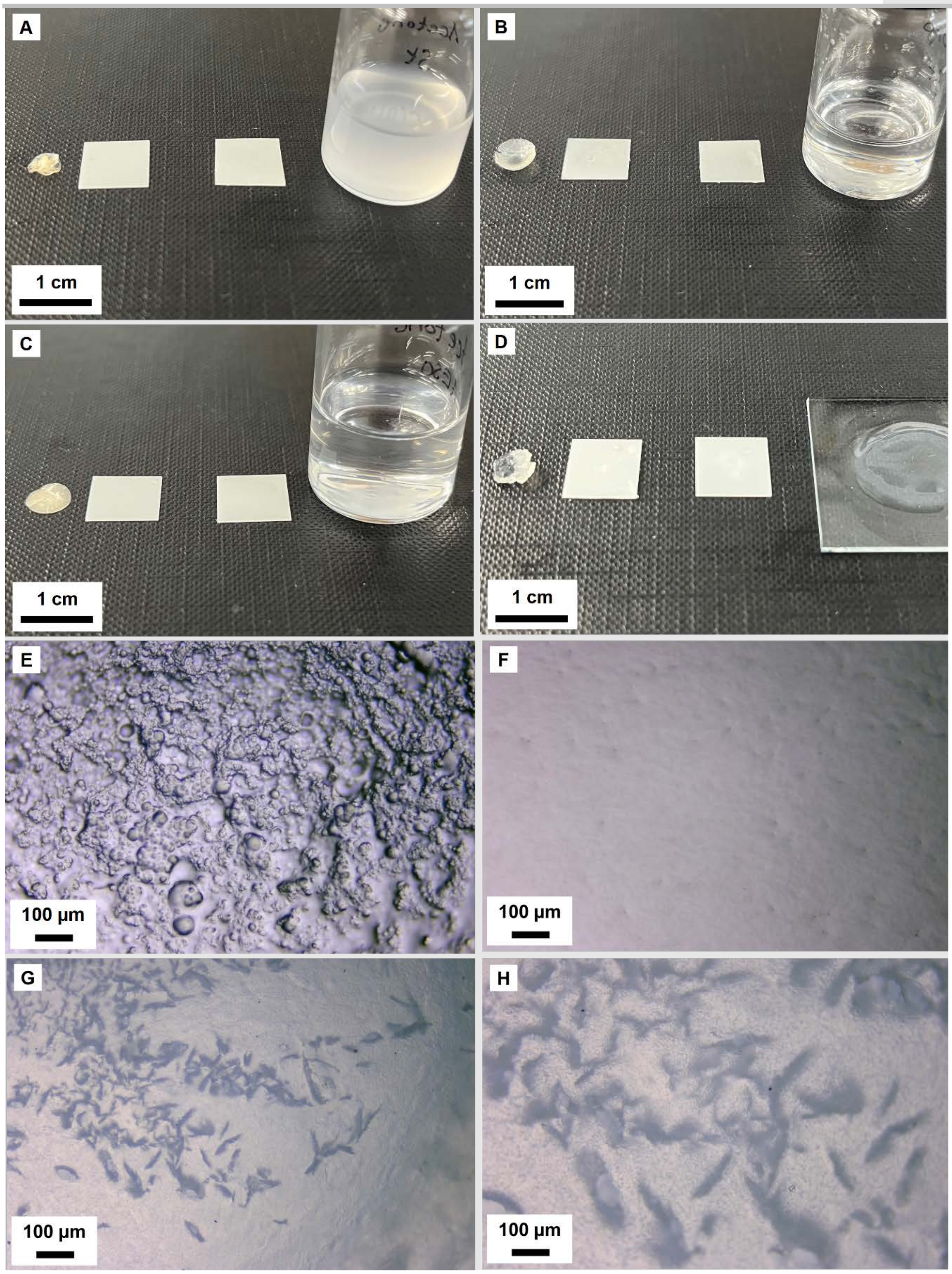

Figure S10. Representative images of stability tests of individual films. A) P(St-BA)/Chi (left) and P(St-BA)/PAA (right) in acetone; B) P(St-BA)/Chi (left) and P(St-BA)/PAA (right) in turpentine; C) P(AESO-BA)/Chi (left) and P(AESO-BA)/PAA (right) in acetone; D) P(AESO-BA)/Chi (left) and P(AESO-BA)/PAA (right) in turpentine; E) Optical micrograph of as-prepared P(AESO-BA)/Chi film; F) Optical micrograph of as-prepared P(AESO-BA)/PAA film; G) Optical micrograph of P(St-BA)/PAA film after exposed to ethanol; H) Optical micrograph of P(AESO-BA)/PAA film after exposed to ethanol.

Reference

[1] R. N. Shimizu, N. R. Demarquette, *J. Appl. Polym. Sci.* **2000**, *76*, 1831–1845.

Author Contributions

M.G. developed the concept. M.G., K.N., and A.S.-R. designed the study. A.S.-R. conducted the experimental work, collected, validated, and analysed data, produced visual material, and wrote the manuscript draft. Laboratory supervision was by K.N. and M.G. Project management was by M.G. and K.N., who also reviewed and edited the manuscript draft, and obtained the funding.
